# Implementing early warning systems for sepsis recognition in African healthcare settings: an Angolan perspective

**DOI:** 10.1016/j.afjem.2026.101001

**Published:** 2026-08-13

**Authors:** Esmael Tomás, Ndenga Tomás

**Affiliations:** a Faculty of Medicine, Agostinho Neto University, Angola; b Clínica Sagrada Esperança, Luanda, Angola; c NOVA National School of Public Health, Comprehensive Health Research Center (CHRC), NOVA University Lisbon, Portugal – Av. Padre Cruz, 1600-560, Lisboa, Portugal

**Keywords:** Sepsis, Early warning score, Emergency medicine, Resource-limited settings, Africa South of the Sahara, Angola

## Abstract

Sepsis remains a major cause of preventable mortality, particularly in low- and middle-income countries, where delayed recognition of clinical deterioration continues to contribute substantially to poor outcomes. The *2026 Surviving Sepsis Campaign* guidelines reaffirm that early identification and timely intervention are key determinants of survival. However, implementing these recommendations in resource-limited African healthcare settings remains challenging. Early Warning Scores, including the Modified Early Warning Score (MEWS), National Early Warning Score (NEWS) and NEWS2, provide simple bedside methods for detecting physiological deterioration using routinely collected vital signs. In many African healthcare environments, workforce shortages, limited monitoring capacity, overcrowding and constrained escalation pathways present important challenges to the reliable use of deterioration-recognition systems. Drawing on emerging African evidence, this commentary examines the opportunities and challenges associated with implementing Early Warning Systems for sepsis recognition in African emergency and acute care settings. We argue that the principal challenge is not modification of existing physiological scoring systems, but rather their sustainable integration into routine clinical practice. Future efforts should prioritise external validation studies, pragmatic health-systems research, workforce training and African-led research collaborations to strengthen the evidence base supporting Early Warning Systems across diverse healthcare environments. Improving sepsis outcomes in Africa will depend not only on recognising deterioration earlier, but also on ensuring that healthcare systems can respond effectively when deterioration is detected.

## African relevance

• Sepsis remains a leading cause of preventable mortality across Africa, where delayed recognition of clinical deterioration contributes substantially to poor outcomes.
• Early Warning Systems provide pragmatic bedside tools that can support earlier recognition and timely escalation of suspected sepsis using routinely collected physiological observations.
• The greatest challenge in African healthcare settings is not adapting physiological scoring systems but integrating them into routine clinical practice through workforce training, structured escalation pathways and context-appropriate implementation strategies.
• African implementation experience demonstrates that low-cost, scalable approaches to deterioration recognition can strengthen patient safety and may inform sepsis care in other resource-constrained health systems worldwide.

## Introduction

Sepsis remains one of the leading causes of critical illness and in-hospital mortality worldwide, disproportionately affecting low- and middle-income countries (LMICs), particularly across sub-Saharan Africa, where healthcare systems continue to develop under significant workforce and infrastructure constraints [[1], [2], [3], [4]]. Delayed recognition of clinical deterioration, limited access to intensive care resources, shortages of trained personnel, and inconsistent physiological monitoring continue to contribute to avoidable morbidity and mortality [[4], [5], [6], [7]].

The publication of the *Surviving Sepsis Campaign: International Guidelines for Management of Sepsis and Septic Shock 2026* further reinforces the central role of early identification, timely escalation, and rapid therapeutic intervention in improving survival [1]. These recommendations are especially relevant to African emergency departments, where patients often present late, emergency units are overcrowded and monitoring resources are limited [3,8,9]. Under these circumstances, pragmatic approaches that support rapid recognition of deterioration may substantially improve patient outcomes [5,8].

Early Warning Scores (EWS) provide a structured method for identifying physiological deterioration using routinely collected vital signs. However, an important question remains: how can early recognition strategies be operationalised effectively in healthcare systems with major structural limitations?

Across many African hospitals, high patient volumes, limited staffing and intermittent physiological monitoring restrict the timely recognition and escalation of clinical deterioration [[5], [6], [7]]. Consequently, the success of sepsis recognition strategies depends not only on clinical knowledge, but also on whether deterioration-recognition systems can be implemented reliably within routine clinical workflows [[5], [6], [7]].

This commentary examines how EWS can be integrated into routine emergency and acute care across diverse African healthcare settings, drawing on emerging evidence and lessons from Angola to identify practical approaches for strengthening early recognition of sepsis.

## Early warning scores as tools for early sepsis recognition

Over the last two decades, EWS have become increasingly integrated into hospital safety frameworks worldwide [[10], [11], [12], [13]]. Tools such as the Modified Early Warning Score (MEWS), National Early Warning Score (NEWS), and NEWS2 aggregate routinely measured physiological parameters to identify patients at risk of adverse clinical outcomes [[10], [11], [12]].

Importantly, these tools are bedside physiological scoring systems that do not require laboratory investigations and can be applied using routinely collected vital-sign observations. Effective use of EWS requires consistent physiological observations, accurate score calculation and timely escalation of abnormal findings.

Within high-income healthcare systems, EWS are commonly linked to rapid response systems, escalation protocols and multidisciplinary deterioration pathways, facilitating earlier recognition of physiological decline, improved communication among healthcare professionals and timely escalation of care [10,13]. Since sepsis frequently presents initially through subtle physiological abnormalities, EWS may also function as practical screening instruments capable of supporting earlier identification of septic patients before the onset of irreversible organ dysfunction [[14], [15], [16]].

Although most EWS were developed and validated predominantly in resource-rich healthcare environments, increasing evidence suggests that they can provide clinically useful prognostic information in LMICs [[10], [11], [12]]. Recent systematic evidence on the performance of EWS in LMICs suggests that these tools generally demonstrate moderate to good discrimination for predicting mortality and adverse events, but their performance remains heterogeneous across settings [5,17]. This variability likely reflects differences in healthcare infrastructure, patient populations, escalation capacity, and local implementation [5,7].

These findings indicate that introducing Early Warning Systems into African healthcare settings should be supported by local validation, assessment of predictive performance and context-sensitive implementation strategies rather than assumptions that performance will automatically translate across healthcare environments.

**Fig. 1:**
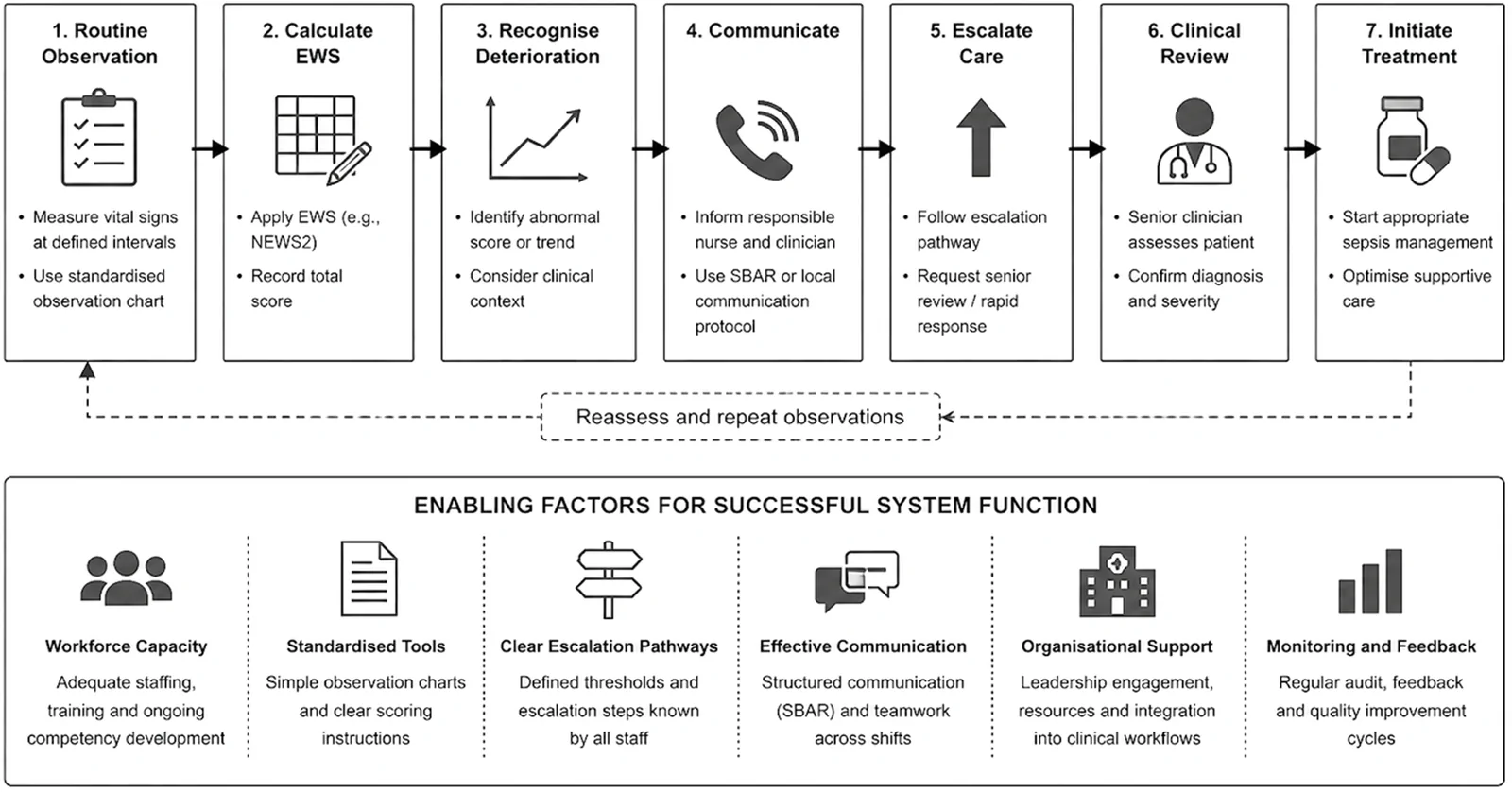
**Conceptual pathway for Early Warning Score–guided recognition and escalation of suspected sepsis**. Early Warning Scores pathway for recognition of clinical deterioration and escalation of care in resource-constrained African healthcare settings. Effective use depends on structured physiological observations, prompt communication, senior clinical review and initiation of appropriate sepsis management. EWS = Early Warning Score; NEWS2 = National Early Warning Score 2; SBAR = Situation, Background, Assessment, Recommendation.

The practical pathway through which EWS support early recognition and escalation of care in resource-constrained healthcare settings is summarised in Fig. 1.

## Operational realities and implementation challenges

Across Africa, healthcare systems operate under operational realities that differ substantially from those of the environments where most early warning systems were initially developed [[5], [6], [7]]. High nurse-to-patient ratios, inconsistent monitoring, limited access to pulse oximetry and observation charts, and the continued reliance on paper-based documentation all influence the feasibility of implementing structured deterioration-recognition systems [[5], [6], [7]].

Under these circumstances, the effectiveness of an early warning system depends not solely on its predictive accuracy, but also on its usability [5,10,18]. Even simple physiological scoring systems may be difficult to implement consistently in high-workload environments when staffing levels are limited, monitoring schedules are difficult to maintain, or escalation processes are poorly defined [5,7,18]. Similarly, protocols that assume immediate access to rapid response teams or intensive care beds may not align with local care structures [5,7].

Consequently, practical approaches in LMICs increasingly emphasise local validation, adaptation to local healthcare context and ongoing evaluation after adoption [5,7,19,20]. These strategies may include linguistic adaptation of supporting materials, simplification of escalation protocols, adaptation to available monitoring capacity, integration into paper-based documentation systems, and targeted staff education and implementation support.

Table 1 summarises practical strategies that may facilitate implementation in resource-constrained hospitals.

**Table 1:** Practical approaches to Early Warning Scores implementation in resource-constrained African hospitals.

Implementation challenge	Why it matters	Potential solution
Staff shortages	Delays in observation and response	Tiered escalation pathways
Limited monitoring	Missed deterioration	Risk-based observation frequency
Lack of rapid response teams	Incomplete physiological assessment	Senior clinician trigger systems
Equipment shortages	Inconsistent score calculation	Paper-based charts
Training gaps	Delayed escalation	Structured competency programmes

Simple colour-coded observation charts linked to predefined escalation actions may represent feasible alternatives in hospitals where electronic monitoring systems and rapid response teams are unavailable. Likewise, integrating EWS into emergency triage workflows may enable earlier identification of high-risk septic patients at the point of first clinical contact.

Overall, these findings reinforce the view that EWS are not merely mathematical scores, but components of broader patient safety systems.

## Examples of African implementation experiences

Although evidence remains limited, several African initiatives illustrate how EWS can be introduced within resource-constrained healthcare environments.

Angola provides an illustrative example. The cross-cultural adaptation of NEWS2 into Angolan Portuguese improved the linguistic clarity and contextual usability of the tool without altering the physiological variables or scoring thresholds [19]. In Angola, efforts to integrate structured deterioration-recognition systems are taking place in hospitals where paper-based observation charts remain common, nurse-to-patient ratios vary considerably, continuous physiological monitoring is often unavailable outside critical care, and rapid response teams are uncommon. Consequently, successful integration depends not only on the score itself but also on simple observation tools, clear escalation pathways, workforce training and organisational support. Additional work from Angola has explored consensus-based approaches to identifying priorities for deterioration recognition and response within local healthcare systems [20]. Rather than propose a new physiological score, the study highlighted the importance of operational factors such as workforce capacity, escalation procedures and staff education, reinforcing the importance of integrating EWS into routine clinical practice.

Evidence from sub-Saharan Africa suggests that existing EWS can demonstrate useful predictive performance, although further multicentre validation studies are required before considering model updating or recalibration [21,22]. Such work is essential because prognostic models should not be assumed to perform identically across different healthcare environments. Qualitative implementation research has further identified barriers, including workload pressures, inconsistent monitoring practices, equipment availability and organisational support [21]. Collectively, these strategies reinforce the principle that Early Warning Systems should be regarded as complex health-system interventions rather than isolated scoring tools. Lessons from Angola and other African countries demonstrate that adapting clinical workflows, workforce training and escalation pathways can facilitate successful integration of EWS into routine care.

## Early warning scores as health-system interventions: lessons from Africa

Successful implementation of any track-and-trigger system depends on several complementary components, including accurate measurement and documentation of physiological observations, timely recognition of abnormal scores, clear escalation pathways, staff confidence in activating escalation systems, availability of senior clinical review, and an organisational culture that supports patient safety. Weakness in any of those elements may substantially reduce the effectiveness of the scoring system itself [10,18].

These principles are particularly relevant across African healthcare systems, where acute care pathways are frequently evolving within environments of constrained resources [5,7,8]. Yet these constraints may also create opportunities for innovation, simplification, and scalable redesign. In resource-constrained healthcare settings, simplicity may represent a strength rather than a limitation. Low-cost paper-based observation charts, simplified escalation algorithms, mobile communication strategies, and tiered response models may all offer practical alternatives capable of improving early sepsis recognition without requiring high-cost technological investment [7,10,19,20].

Because many African hospitals are not constrained by legacy electronic infrastructures or rigid historical models, there may be greater flexibility to design pragmatic, locally adapted deterioration recognition systems from the outset [7,19,20].

Importantly, African experience offers lessons that extend beyond the continent. Resource constraints have encouraged innovation, simplification and pragmatic redesign, generating practical approaches that may also benefit healthcare systems with limited resources elsewhere. By demonstrating how EWS can be integrated into routine practice using low-cost, scalable approaches, African programmes contribute valuable evidence to the broader global effort to improve patient safety and sepsis recognition.

## Implications for global sepsis strategies

The *2026 Surviving Sepsis Campaign* has played a transformative role in shaping international approaches to sepsis management [1]. However, future global sepsis initiatives may benefit from stronger emphasis on health-system research and contextual adaptability.

International recommendations should increasingly acknowledge that early recognition systems must function across highly variable healthcare environments. Rather than assuming uniform operational capacity, future guidance could incorporate adaptable implementation frameworks that recognise differing levels of monitoring infrastructure, workforce availability, and escalation resources.

Without context-sensitive implementation strategies, the benefits of global sepsis recommendations may not be realised equally across all healthcare environments, potentially widening existing disparities in timely recognition and treatment. Priorities for future work should therefore include multicentre validation studies in LMIC populations, pragmatic health-systems research, workforce education and sustainable systems for monitoring implementation and clinical performance.

Contextual adaptation should not be mistaken for reduced standards of care. Rather, it reflects the principle that effective patient safety interventions must align with operational reality if they are to achieve meaningful clinical impact. As this commentary does not present new empirical data and its conclusions are based on interpretation of the available literature, the priority actions discussed should be viewed as areas for future research, evaluation and policy development rather than definitive recommendations. Further multicentre implementation studies across diverse African healthcare settings are needed to strengthen the evidence base.

## Conclusion

Sepsis remains a major cause of preventable mortality across Africa. EWS provide a practical approach to recognising clinical deterioration, but their impact depends on reliable integration into routine care through structured observation and timely escalation. Emerging evidence from African healthcare demonstrates that meaningful progress is achievable through locally adapted clinical workflows, workforce education and stronger organisational support. These experiences suggest that improving sepsis outcomes will require sustained investment not only in critical care capacity but also in frontline emergency and acute care services that can recognise deterioration early and respond effectively. Ultimately, improving sepsis outcomes in Africa will depend not on developing increasingly sophisticated EWS, but on embedding deterioration-recognition systems that frontline clinicians can apply consistently within routine care.

## Declarations

### Declaration of generative AI and AI-assisted technologies in the manuscript preparation process

During the preparation of this work, the authors used the Large Language Model ChatGPT (OpenAI, GPT-5.5) to assist with language editing and the development of the conceptual figure. All AI-assisted output was critically reviewed, revised and verified by the authors, who take full responsibility for the accuracy, integrity and originality of the published article.

### Dissemination of results

The findings and perspectives presented in this commentary will be disseminated through scientific meetings, and professional networks involved in emergency, acute, and critical care across Africa.

## Funding

No external funding was received.

## CRediT authorship contribution statement

**Esmael Tomás:** Conceptualization, Methodology, Investigation, Writing – original draft, Writing – review & editing. **Ndenga Tomás:** Writing – review & editing, Supervision.

## Declaration of competing interest

The authors declare no competing interests.
